# Hospital acquired Acute Kidney Injury is associated with increased mortality but not increased readmission rates in a UK acute hospital

**DOI:** 10.1186/s12882-017-0729-9

**Published:** 2017-10-20

**Authors:** Nerissa Jurawan, Tanya Pankhurst, Charles Ferro, Peter Nightingale, Jamie Coleman, David Rosser, Simon Ball

**Affiliations:** University Hospitals Birmingham, Birmingham, UK

## Abstract

**Background:**

Acute Kidney Injury (AKI) has evoked much interest over the past decade and is reported to be associated with high inpatient mortality. Preventable death and increased readmission rates related to AKI have been the focus of considerable interest.

**Methods:**

We studied hospital acquired AKI in all emergency hospital admissions, except transfers from ICU to ICU or patients known to renal services, to ascertain mortality and readmission rates, and trackable modifiable factors for death, using cox regression and Kaplan Meier survival curves. Data was extracted from the electronic patient records and a series of case notes reviewed. Admissions were included between April 2006 and March 2010 (and patients followed up until September 2011).

**Results:**

Overall incidence of AKI was 2.2%, (AKI stage 1, 61%, stage 2,27% and stage 3, 12%). In patients who sustain in-hospital AKI, 34% die in hospital, 42% are dead at 90 days and 48% at 1 year post discharge, compared to 12% 1 year mortality in patients without AKI. In multivariable analyses, AKI is an independent risk factor for in-hospital mortality (Hazard Ratio 1.6: 95% confidence intervals 1.43–1.75: *P* < 0.001), death within 90 days of discharge (Hazard Ratio 1.5: 95% confidence intervals 1.3–1.9: P < 0.001) and subsequent mortality beyond 90 days (Hazard Ratio 2.9: 95% confidence intervals 2.7–3.1: *P* < 0.001) after adjustment for co-morbidities and peak C-reactive protein.

Thirty percent of the patients who died in the first 90 days post discharge and had AKI, also had malignancy. Readmission rates at 30 and 90 days were not increased by AKI after adjustment for co-morbidities and peak C-reactive protein.

**Conclusions:**

A significant proportion of deaths in the first 90 days post-discharge may not be avoidable, due to malignancy and other end-stage disease. Readmission rates were not higher in patients who had had AKI.

**Electronic supplementary material:**

The online version of this article (10.1186/s12882-017-0729-9) contains supplementary material, which is available to authorized users.

## Background

Acute Kidney Injury (AKI) is a frequent complication of acute illnesses requiring hospitalisation, affecting approximately 10% of patients [1] depending on how it is measured. It is associated with increased morbidity, a doubling of length of stay (LoS) in hospital [2, 3] and an excess in-hospital mortality 6–9 times greater than patients without AKI, independent of established co-morbidities [4, 5]. Episodes of AKI have also been associated with increasing risks of chronic kidney disease (CKD), stroke and other cardiovascular events [6, 7].

The data on mortality after surviving an episode of AKI in unselected inpatient population studies, is limited [8, 9]. A study from the US Veterans Administration reported that excess mortality risk persists after discharge and at 1 year is double that of patients without AKI [1]. However, 95.1% of this cohort was male. Other studies have generally been limited in size and to specific disease settings [10–14]. The data on readmission rates after an episode of AKI is even more limited with studies largely in specific diseases, showing increased readmission rates [4, 15–18]. To the best of our knowledge there is no large scale study examining hospital readmission rates from European healthcare systems.

With the increasingly widespread use of electronic patient records (EPR), it may be possible to identify patients with, or at risk of, AKI as well as associated and potentially reversible factors [19]. We have previously shown that systemic inflammation, as determined by C-reactive protein (CRP), is a major determinant of AKI [20]. To date no study has examined the relationship between in-hospital CRP and outcomes post-AKI.

Acute kidney injury is expensive [2] and potentially preventable [21]. It is therefore a legitimate target for modification relevant to both improved patient and health economic outcomes. Despite this, few patients are subject to specialist review following discharge. There is a need for evidence to inform whether targeted post-discharge interventions based upon a history of AKI would add value to patient care. This includes evidence regarding readmission and mortality within the first 90 days post-discharge, as arguably this is a period during which intervention might reasonably be focussed. This would have resource implications and evidence from a contemporary European population is needed to assess whether intervention might reasonably be expected to influence outcome.

Our principle questions were therefore whether AKI in patients not previously followed by a nephrologist, had similarly high rates of deaths associated with AKI and whether there were identifiable avoidable deaths. We also wanted to understand whether readmission rates in survivors were higher in patients with AKI where targeted resource allocation may be helpful.

We, therefore, analysed a large, unselected population in a single UK centre looking at emergency admissions, in conjunction with parameters available from the EPR to determine in-hospital mortality, as well as readmission rates and mortality in the year post discharge after an episode of AKI. We also examined the causes of death, including malignancy within 90-days of discharge in patients who had suffered an episode of AKI.

## Methods

We studied all adult emergency admissions to an urban, 1200 bedded tertiary academic referral centre in the UK, between April 2006 and March 2010. The institution admits approximately 42,000 emergency admissions per year. It is the regional trauma centre and has 100 Intensive Care (ICU) beds; it does not have paediatric or obstetric and gynaecology services.

Hospital acquired AKI was studied defined on first and highest creatinines after admission by the proportionate KDIGO classification, parameters that should be available in all UK hospitals. Baseline kidney function, which is currently unknown in many emergency admissions in the UK, was not included in this study. AKI was defined as a delta change from the first creatinine taken in the emergency admission. In addition to patients who did not have AKI on creatinine measurement, patients were assumed to have no AKI if they had no creatinine or a single creatinine measured.

**Fig. 1:**
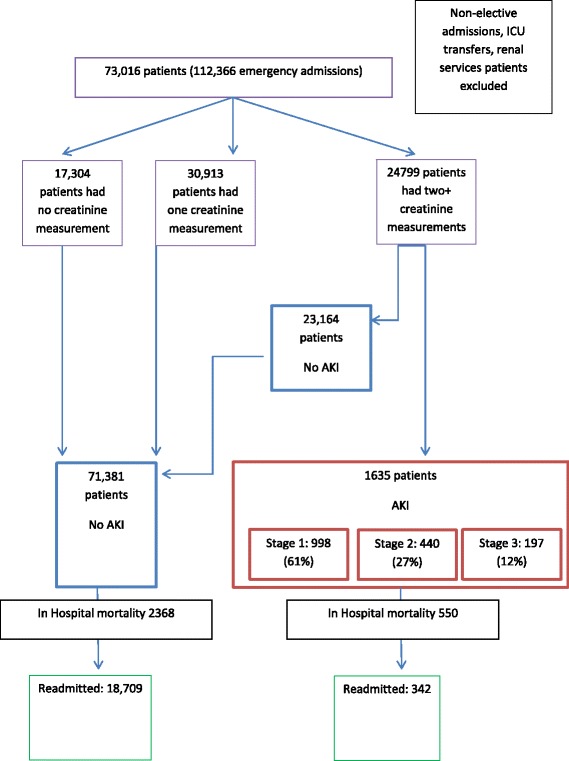
Flow diagram of patient included in the study

All emergency admissions were included except where patients were previously known to the renal service, or were transferred directly into ICU from another centre (Fig. 1). Patients known to nephrologists were excluded on the basis that their higher risk for AKI is known and their care already under renal specialists. Readmission and admission data was analysed for emergency admission only, no elective admission data was included.

Data collated from EPR included: demography, time-stamped drug administration (Additional file 1), ICD-10 diagnoses coded at discharge (for definitions of all diagnoses via ICD-10 see Additional file 2, infection is an aggregate of several ICD-10 codes), routine biochemical analyses, parenteral radiological contrast administration, the need for renal replacement therapy (RRT), length of stay, death in hospital and post-discharge (to census date 30/9/2011). Readmission data was extracted from the hospital systems (from Lorenzo, the Patient Administration System) by the Informatics Department, this data is used to report hospital activity nationally. Data on patient death was extracted from the NHS Spine (national death files), also via Informatics.

The timeframe for data collection was 24 h prior to admission until discharge, except in those meeting the criteria for hospital-acquired AKI in which case the C-reactive protein concentration (CRP) was censored on the day of the peak creatinine. CRP is not adjusted for renal function in our laboratories. Creatinines were measured using a Jaffe method on a Cobas 8000 machine with calibration traceable to isotope dilution mass spectrometry. Patient survival and readmission was analysed following an index admission. The index admission was the first in the study period for all patients. Time to readmission was analysed from both admission and discharge date to readmission (in separate analyses – available on request). CRP was stratified in deciles to 100 mg/dL after which it was stratified in 50 mg/dL up to 400 mg/dL, based on prior clinic consensus and previous published data. This ensured adequate event rates and numbers of patients per stratum [20, 22].

Using data within the EPR, we subsequently examined subsets of patients who had AKI in greater detail. In order to establish whether there were any common factors amenable to manipulation to reduce the mortality in the patients dying in the first 90 days post discharge, we reviewed the case notes of 153 cases of patients with AKI, manually. Cases were reviewed by three experienced clinicians using a semi-structured proforma to look for cause of death, diagnosis of AKI and any factors that could have changed outcome as previous NCEPOD reports have done [21].

### Definition of acute kidney injury

The definition for AKI has changed within recent years with interdisciplinary consensus groups proposing standardized systems to define and stage AKI and now internationally agreed in the KDIGO criteria [23]. All of the existing criteria were not only designed to diagnose and assess the severity and progression of AKI but also to provide some prediction of mortality. We defined AKI from the KDIGO definition, using the proportionate values only. Specifically AKI Stage 1 was defined as a serum creatinine increase of 1.5–1.9 times index or ≥0.3 mg/dL; AKI Stage 2 as a serum creatinine increase of 2.0–2.9 times index and AKI Stage 3 as a serum creatinine increase of ≥3.0 times index creatinine or ≥4.0 mg/dL or initiation of RRT.

### Data and statistical analyses

We used hospital data from the central EPR at our institution. Data analysts extracted and anonymised data from the central EPR into databases for analysis, patient identifiable data was not included; IRB approval was therefore not requested. The EPR, (Patient Information and Communication System, PICS) is a commercially available EPR. Data extraction from the system is robust and supports all national clinical reports; the informatics team also build international reporting tools for hospital episode statistics.

Statistical analyses were undertaken using Minitab v15 (Minitab Inc., State College, Pa., USA) and SPSS (Version 22, Chicago, Ill, USA). AKI and in-hospital mortality were treated as events associated with a specific admission independent of time and one another. Continuous variables are described as mean and standard deviation (SD) or median with interquartile range (IQR) and the significance of differences quantified by Student’s t test or Mann Whitney-U test as appropriate. Categorical variables are described as proportions and compared by χ2 test.

We then used time dependent cox regression to look at the relationship between AKI, mortality and co-morbidities for inpatient and post discharge death after 90 days, taking into account the development of AKI in subsequent readmissions following the index admission. Results are presented as hazard ratio (HR) with 95% confidence intervals (CI). Proportional hazards assumption was checked and found appropriate for the Cox regression models. Kaplan Meier curves were used for survival analyses. Deaths were analysed on index admission only.

Comorbidities tested for in the model were diabetes mellitus, hypertension, ischaemic heart disease, heart failure, vascular disease, malignancy, composite of infection (see appendices for these codes), liver disease, and composite of gastro-intestinal blood loss or hypovolemia.

We performed sensitivity analyses to establish that hazard ratios were not changed if creatinines peaking before 48 h were excluded, in order to ensure community acquired AKI was not included in the analysis.

We also looked at patients who specifically had malignancy, and what percentage of these patients died in the index admission and in the subsequent 90 days. We looked at the proportions of these patients with and without AKI and their median admission creatinine.

Crude readmission rates, not taking into account deaths, were calculated at 30 and 90 days post-discharge. Additionally, readmission was analysed for 30 day and 90 day readmission rates using logistic regression. We performed these analyses both including and excluding patients dying within these time frames (further results in additional files 1, 2, 3, 4, 5, 6, 7, 8, 9, 10 and 11). Time to first readmission was analysed, censored for death, by cox regression, for time from index admission discharge and time from admission. Number of readmissions was analysed by Mann-Whitney U.

To compare the populations who died in the index admission; or who survived were re-admitted; or who survived and were not readmitted, we compared the co-morbidities of these three populations against each other using χ2 and then Fisher’s Exact test. Kaplan Meier curves were used to analyse readmission from index admission discharge time and from admission time.

## Results

In the 4 year study period, 73,016 individual patients had one or more emergency admissions, in total, 112,366 admissions. Men represented 52.8% of the population and the mean age was 56.8 years (Table 1). No creatinine measurement was available in 15.4% of index admissions, and 30, 913, (42.3%) patients had only one creatinine measured.

**Table 1:** Demographics and mortality for hospitalised patients

	Total	No AKI	AKI	*P* value
Number of patients	73,016	71,381	1635 (2.2%)	
Age/years (median (IQR))	56.8 (38–76)	56.5 (38–76)	71.0 (61–84)	<0.001
Male n (%)	38,568 (52.8)	37,706 (53.0)	862 (53)	NS
LOS/days (mean, IQR))	7.55 (1–8)	7.0 (1–7)	31.1 (11–41)	<0.001
Mortality at discharge (% of whole population)	2918 (3.9)	2368 (3.3)	550 (33.6)	<0.001
Total mortality at 90 Days	5546 (7.6)	4830 (6.8)	689 (42.1)	<0.001
Total mortality at 1 year	9088 (12.4)	8301 (11.6)	787 (48.1)	<0.001
Number of patients readmitted during follow-up (until censor 30/9/2011) (%)	18,971 (26.0)	18,709 (26.2)	342 (21.0)	<0.001
eGFR 1st measurement (ml/min/1.73m^2^) (median (IQR))	76.4 (57.2–92.1)	76.5 (57.5–92.2)	73.2 (45–88.5)	<0.001
eGFR minimum (ml/min/1.73m^2^) (median (IQR))	73.1 (54.3–89.2)	74.1 (55.6–89.8)	33.3 (18.0–41.5)	<0.001
eGFR on 1st measurement <45/ml/min/1.73m^2^ (%)	7281 (10.0)	6872 (9.6)	409 (25.0)	<0.001
eGFR minimum <45/ml/min/1.73m^2^ (%)	4372 (6.0)	4157 (5.8)	215 (13.1)	<0.001
Creatinine on first measurement >177 μmol/L (>2 mg/dL)(%)	2290 (3.1)	2137 (3.0)	153 (9.4)	<0.001
Creatinine minimum >177 μmol/L (>2 mg/dL)(%)	1022 (1.4)	949 (1.3)	73 (4.5)	<0.001
Co-morbidities				
Diabetes mellitus n (%)	7571 (10.4)	7227 (10.0)	344 (21.0)	<0.001
Hypertension n (%)	15,503 (21.2)	14,877 (20.8)	626 (38.3)	<0.001
Ischaemic heart disease n (%)	4001 (5.5)	3832 (5.4)	169 (10.3)	<0.001
Heart failure n (%)	2460 (3.4)	2183 (3.1)	227 (13.9)	<0.001
Vascular disease n (%)	762 (1.0)	715 (1.0)	47 (2.9)	<0.001
Malignancy n (%)	5518 (7.6)	5225 (7.3)	293 (17.9)	<0.001
Composite of Infection n (%)	4920 (6.7)	4457 (6.2)	463 (28.3)	<0.001
Liver Disease n (%)	1351 (1.9)	1167 (1.63)	184 (11.3)	<0.001
Composite of GI blood loss or hypovolemia n (%)	2058 (2.8)	1910 (2.7)	148 (9.1)	<0.001
CRP measurement	30,688 (42)	29,146 (40)	1542 (94)	<0.001
Average CRP (in those who had CRP measured)	88	83	174	<0.001

*AKI*, acute kidney injury, *eGFR* estimated glomerular filtration rate, *IQR* interquartile range, *LOS* length of stay

There were 1635 (2.2%) patients in whom the index admission was associated with an episode of hospital-acquired AKI (stage 1: 998 (61%), stage 2: 440 (27%), stage 3: 197 (12%). Of note many published studies quote AKI incidence as a percentage of the patients who had two or more creatinines measured, in which case the incidence of AKI would be 5.3%. We used the whole population; pragmatically, treating clinicians would have to assume that patients with unmeasured delta renal function did not have AKI.

As expected, patients with AKI were older and had a greater co-morbidity than those without hospital-acquired AKI.

Index admission creatinine measured >177 μmol/L (2 mg/dL) in 2290 (3.1%) of patients and 0.51% had an admission creatinine >354 μmol/L (4 mg/dL).

### Mortality

**Fig. 2:**
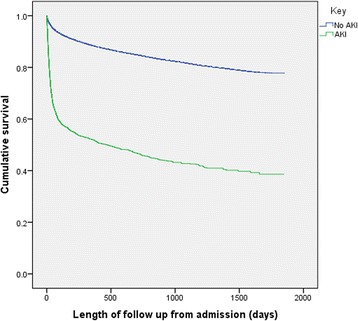
Overall survival of all patients in the study from admission dependent on AKI (*p* = <0.001)

The in-hospital mortality associated with the index admission was 3.9% in those without AKI and 33.6% in those with AKI (Fig. 2).

There were 70,098 patients who survived to leave hospital (69,013 without AKI and 1085 with AKI). By 90 days a further 4% (2462) of those without AKI and 9% (98) of those with AKI had died making the total 90 day mortality for patients without AKI 6.8% and for those with AKI 42.1%. By 1 year, 8301 patients without AKI had died (total no-AKI mortality 11.6%) and 787 of those with AKI had died (total AKI mortality 48.1%). The fully adjusted HR for inpatient mortality associated with AKI was 1.6 (95% CI 1.43–1.75 *p* < 0.001) for all AKI (Table 2); for patients discharged, fully adjusted HR for mortality at 90 days was 1.5 (95% CI 1.3–1.9) (data available on request), increasing with severity of AKI (Table 3) and at 1 year the adjusted hazard ratio for patients with AKI was 2.9 (95% CI 2.7–3.1) (Additional file 6). The survival of patients with and without AKI is shown in Kaplan Meier curves (Fig. 2).

**Table 2:** Cox regression for in-hospital mortality for AKI and adjusted for age, gender, co-morbidity (Diabetes Mellitus; hypertension; heart failure; vascular disease; malignancy; composite of infection; composite of GI blood loss or hypovolemia) and CRP

	Hazard Ratio	95% Confidence Intervals		*P* value
		Lower	Upper	
AKI	1.580	1.428	1.747	<0.001
Male gender	1.025	0.951	1.104	0.516
Age (reference [18–35] years)				
Age 36–45	1.941	1.250	3.013	0.003
Age 46–55	4.097	2.819	5.955	<0.001
Age 56–65	5.517	3.860	7.887	<0.001
Age 66–75	8.650	6.110	12.248	<0.001
Age > 75	16.868	12.028	23.655	<0.001
Diabetes Mellitus	0.960	0.868	1.062	0.425
Hypertension	0.741	0.683	0.803	<0.001
Ischaemic Heart Disease	0.782	0.675	0.906	0.001
Heart Failure	1.695	1.530	1.879	<0.001
Vascular Disease	1.512	1.244	1.839	<0.001
Malignancy	1.884	1.728	2.054	<0.001
Composite of Infection	0.814	0.741	0.895	<0.001
Liver Disease	2.612	2.261	3.017	<0.001
Composite of GI Blood Loss or Hypovolaemia	1.627	1.451	1.823	<0.001
CRP (referenced to CRP < 11)				
Unmeasured	1.217	.904	1.637	0.195
11–20	2.097	1.577	2.788	<0.001
21–30	2.223	1.664	2.969	<0.001
31–40	2.664	2.004	3.542	<0.001
41–50	2.377	1.763	3.204	<0.001
51–60	2.237	1.642	3.050	<0.001
61–70	3.013	2.246	4.043	<0.001
71–80	2.660	1.976	3.579	<0.001
81–90	3.220	2.388	4.343	<0.001
91–100	3.087	2.451	3.889	<0.001
101–150	3.636	2.872	4.605	<0.001
151–200	4.026	3.184	5.091	<0.001
201–250	4.091	3.187	5.251	<0.001
251–300	4.441	3.441	5.731	<0.001
301–350	4.254	3.091	5.853	<0.001
351–400	2.966	2.373	3.707	<0.001
> 400	5.917	4.464	7.843	<0.001

*AKI* acute kidney injury, *CRP* C-reactive protein

**Table 3:** Cox regression for 90 day mortality AKI stage and adjusted for age, gender, co-morbidity (Diabetes Mellitus; hypertension; heart failure; vascular disease; malignancy; composite of infection; composite of GI blood loss or hypovolemia) and CRP

	Hazard Ratio	95% Confidence intervals		*P* value
		Lower	Upper	
AKI stage1	1.257	1.017	1.554	0.034
AKI stage2	1.555	1.119	2.160	0.009
AKI stage 3	2.390	1.612	3.544	<0.001
Male gender	0.938	0.871	1.010	0.091
Age (reference [18–35] years)				
Age 36–45	3.048	2.103	4.415	<0.001
Age 46–55	5.162	3.687	7.228	<0.001
Age 56–65	8.320	6.044	11.454	<0.001
Age 66–75	12.384	9.047	16.953	<0.001
Age > 75	24.881	18.320	33.792	<0.001
Diabetes Mellitus	1.028	0.925	1.143	0.605
Hypertension	0.754	0.693	0.821	<0.001
Ischaemic Heart Disease	0.825	0.704	0.968	0.018
Heart Failure	1.657	1.443	1.903	<0.001
Vascular Disease	1.656	1.289	2.126	<0.001
Malignancy	6.340	5.864	6.855	<0.001
Composite of Infection	1.047	0.932	1.177	0.439
Liver Disease	2.166	1.802	2.604	<0.001
Composite of GI Blood Loss or Hypovolaemia	1.813	1.575	2.086	<0.001
CRP (referenced to CRP < 11)				
Unmeasured	1.496	1.247	1.795	<0.001
11–20	1.504	1.218	1.856	<0.001
21–30	1.902	1.541	2.348	<0.001
31–40	2.108	1.700	2.614	<0.001
41–50	1.837	1.451	2.326	<0.001
51–60	2.557	2.052	3.187	<0.001
61–70	2.827	2.248	3.556	<0.001
71–80	2.411	1.904	3.054	<0.001
81–90	1.994	1.525	2.608	<0.001
91–100	2.585	2.193	3.047	<0.001
101–150	2.369	1.968	2.852	<0.001
151–200	2.421	1.998	2.935	<0.001
201–250	2.238	1.769	2.832	<0.001
251–300	2.359	1.817	3.064	<0.001
301–350	1.725	1.113	2.674	0.015
351–400	0.927	0.801	1.074	0.313
> 400	1.836	1.231	2.740	0.003

*AKI* acute kidney injury, *CRP* C-reactive protein

We performed sensitivity analysis to ensure that HRs were similar for patients when creatinine peaking within 48 h were excluded, to allow for community acquired AKI to be excluded. HR for inpatient mortality for patients with AKI with peak creatinine more than 48 h after admission was similar: 1.82 (CI 1.61–2.07) and for 90 day mortality for AKI stage (see comparison to Table 3) HR were 1.32 (1.05–1.66) for AKI 1; AKI 2 1.46 (1.03–2.09) and AKI 3 2.21 (1.46–3.33), very similar to the analysis where all creatinine post admission was included.

### Mortality and CRP

CRP is strongly associated with mortality (additional file 11) Peak CRP was an independent risk factor for in-hospital mortality in multivariate analysis which includes AKI and co-morbidity (Table 2). This effect of elevated CRP concentration persisted in exploratory analyses of mortality post-discharge. A CRP > 20 ng/ml was associated with an odds of mortality at 90 days of 2.0 but this excess risk disappears at 1 year. It was not so influential in analysis of the whole post-discharge period (See additional material). There was no substantial progression of risk with a rising CRP in mortality post discharge.

### Profile of patients who die with AKI, diagnoses, prognoses and modifiable risk factors

We reviewed the case notes of the 153 patients who sustained hospital-acquired AKI and died in the first 90 days post discharge. 67 (43%) had malignancy; 25 (16%) patients had an infectious illness as part of their index admission and 14 (9%) had a documented acute coronary syndrome or myocardial infarction. Sixty two were being palliated (42 for malignancy and the others for dementia, cardiac or other diagnosis) and 11 had dementia. Of the 153 who died, 35 (23%) were readmitted to our institution.

In the 293 patients with malignancy who sustained hospital-acquired AKI, 134 (45%) died during admission and a further 48 (16%) died in the first 90 days following discharge. The median admission creatinine in this group of patients was 93 (73–121)μmol/L. This is higher than the group with who develop in-hospital AKI but do not have malignancy.

### Readmission

**Fig. 3:**
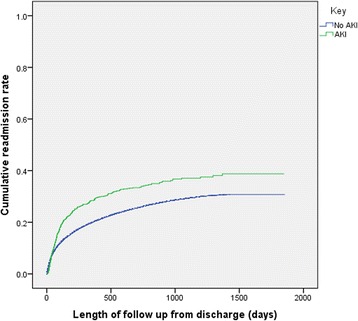
Readmission of all patients in the study from index admission to readmission, end of follow up or death (*p* = <0.001)

Kaplan Meier curves for readmission (Fig. 3) demonstrated in unadjusted readmission, patients with AKI were more likely to be readmitted (log rank p = <0.001). In univariate analysis, previous AKI was associated with an increased risk of readmission (HR 1.5; 95% CI 1.2–1.7; *P* < 0.001). However, after adjustment for sex, age, co-morbidities and peak CRP, previous AKI was no longer associated with an increased risk of readmission.

In adjusted 30 day readmission rate in patients who had suffered AKI, readmission rates were actually lower in patients with AKI (54 (3.3%) than without AKI 4245 (5.9%) *p* = 0.01). For 90 day readmission (Table 4), 7705 (11.1%) of 69,013 patients without AKI, surviving the index admission, were re-admitted and 169 (15.5%) of 1085 surviving patients with AKI were subsequently readmitted (*p* = 0.37).

**Table 4:** Cox regression for readmission within 90 days for AKI or no AKI and adjusted for age, gender, co-morbidity (Diabetes Mellitus; hypertension; heart failure; vascular disease; malignancy; composite of infection; composite of GI blood loss or hypovolemia) and CRP

	Hazard Ratio	95% Confidence intervals		*P* value
		Lower	Upper	
AKI	0.921	0.787	1.078	0.304
Male gender	0.932	0.891	0.974	0.002
Age (reference [18–35] years)				
Age 36–45	1.076	0.983	1.178	0.113
Age 46–55	1.254	1.151	1.366	<0.001
Age 56–65	1.261	1.159	1.374	<0.001
Age 66–75	1.441	1.325	1.567	<0.001
Age > 75	1.784	1.656	1.921	<0.001
Diabetes Mellitus	1.186	1.108	1.269	<0.001
Hypertension	0.939	0.888	0.994	0.029
Ischaemic Heart Disease	1.107	1.011	1.212	0.028
Heart Failure	1.372	1.234	1.527	0.000
Vascular Disease	1.194	0.983	1.450	0.073
Malignancy	2.112	1.975	2.258	<0.001
Composite of Infection	1.149	1.057	1.249	0.001
Liver Disease	1.776	1.556	2.027	<0.001
Composite of GI Blood Loss or Hypovolaemia	1.259	1.116	1.420	<0.001
CRP (referenced to CRP < 11)				
Unmeasured	1.010	0.915	1.115	0.848
11–20	1.069	0.950	1.204	0.267
21–30	1.051	0.920	1.200	0.463
31–40	1.107	0.960	1.276	0.163
41–50	1.139	0.983	1.320	0.084
51–60	1.092	0.931	1.282	0.279
61–70	1.137	0.962	1.344	0.132
71–80	1.069	0.899	1.272	0.450
81–90	.930	0.762	1.134	0.471
91–100	1.068	0.957	1.191	0.238
101–150	1.087	0.957	1.235	0.199
151–200	1.096	0.957	1.255	0.186
201–250	1.088	0.918	1.290	0.332
251–300	0.763	0.611	0.954	0.018
301–350	1.327	1.020	1.726	0.035
351–400	0.843	0.788	0.902	<0.001
> 400	1.042	0.788	1.378	0.773

*AKI* acute kidney injury, *CRP* C-reactive protein

In sensitivity analysis including creatinine before 48 h of admission, AKI remained an insignificant factor for readmission.

## Discussion

There is considerable concern about AKI, and increasingly policy guidelines suggest this is frequent, increasing in incidence and a cause of avoidable death. From the current literature it is also expensive [2] and is associated with high mortality. The actual incidence of AKI is difficult to assess in the UK until there are robust electronic patient records (EPR) in all UK hospitals. Data from the Hospital Episode Statistics (HES) database [2], report the incidence of AKI to be 2.43% but others report this to be much higher [24, 25]. The data presented in this paper, where AKI was identified using electronic systems to detect proportional changes in creatinine, conclude the incidence on admission in a large urban hospital, to be 2.2% (or 5.3% of the population where renal function was measured more than once). We included patients who had only one, or no creatinines measured, in the patients without AKI as these patients would not be identified as having AKI in hospital. Patients known to renal services were also excluded on the basis that they were already under nephrology care and therefore renal risk factors were likely to be closely managed in this group.

Mortality associated with AKI is high compared to a population without AKI and this has been previously documented for in-hospital mortality [4, 5]. In this study in-hospital mortality associated with AKI, was 10 times that of the non-AKI population and worsened with severity of kidney injury. Thirty-three percent of patients who had AKI, died during their hospital admission.

This association between mortality and AKI has previously been reported to persist after discharge [26–28]. Dialysis dependent AKI is associated with increased mortality. There are a few studies which report increased mortality after discharge in specific disease states [28–32] and after ICU admission [33, 34]. A recent study looked at this in a multivariable model for survival post 90 days [1]. They found an adjusted mortality risk of 1.41 for patients who had had AKI not requiring dialysis, and this trend persisted to 4 years follow up.

There are few reports looking at mortality after AKI in unselected hospital populations in the first 90 days post discharge [8, 9] although some studies look at specific patient groups post discharge. In the LaFrance study [1] the risk of dying did persist after discharge and was high within the first 90 days. 42% of patients diagnosed with AKI in hospital had died by 90 days post discharge. Adjusted mortality risk in our study up to 90 days post discharge, was 1.5 for patients who had had inpatient AKI compared to those who did not have AKI. As in previous literature, this mortality risk persists beyond 90 days and up to 1 year [35]. In our data the association between AKI identified in hospital and death was persistent after 90 days follow up.

Risk of dying increased with severity of AKI in hospital, in the first 90 days and post 90 days discharge and this is consistent with previous studies [36–38].

Acute kidney injury in this data was an independent factor for death when adjusted for co-morbidities. The current assumption is that AKI is causative in the high mortality rates with which it is associated, and has attracted considerable attention as being a reversible factor, that is preventable, with lives saved as a result [39]. However, the current NICE AKI guideline states that “No studies were identified for prognostic outcomes with KDIGO for adults, children or young people." [29]. In our study we found that high CRP was an important predictor of death and when combined with a diagnosis of AKI increased the odds ratio of death in hospital to more than 10 times that of patients without AKI. Why AKI and specifically AKI with inflammatory states might be an independent risk factor for death is likely to depend on the effects that these changes have on the entire body state, and experimental models already show examples of changes in the heart and lungs [40–42]. The CRP may represent risk in AKI over and above its association with SIRS/SEPSIS, but as far as we are aware there is no current literature examining the relationship between AKI, CRP and mortality. What is clear from this data is that the combination of high CRP in the setting of AKI greatly increases mortality risk.

We were also interested in defining mortality within the first 90 days as a potential timeframe within which it may be possible to target quality improvement based upon early post-discharge review. Of those 1085 with hospital-acquired AKI who survived to discharge, 13.9% died in the first 90 days post discharge and 22.8% in the first year. Age and malignancy were dominant risk factors for early post discharge mortality in this population. The presence of malignancy, specifically advanced malignancy established by case note review was present in >40% of deaths in this group of patients.

These findings suggest that a simple approach targeting those with hospital-acquired AKI for intervention to prevent early mortality, may have limited utility. Any such intervention would need to account particularly for patients with advanced malignancy. Although this is a single centre study there is a wide range of disease represented and there was no preponderance of patients with a diagnosed malignancy to suggest that the current analysis is particularly unbalanced. This is an important finding in relation to the debate about reduction in death and morbidity due to AKI. There may be utility in understanding the combination of AKI and incurable disease which may serve to target therapies in palliation but it is unlikely that this will reduce overall mortality. This is not to say of course that the presence of malignancy per se means AKI follow-up is unhelpful, patients may have curable cancers and nephrology involvement may influence chemotherapy treatments. In order to target patients where early intervention will reduce mortality related to AKI, it is likely that that this much smaller subset is easier to define as we understand the description of the larger group. The preventable deaths are hidden within this larger subset.

Thirty day readmission is a traditionally collected measure and represents ‘failed discharges’. In terms of health economics, and resource, readmissions alone, independent of deaths, are an important resource model. In this study in cox regression, diabetes, ischaemic heart disease, heart failure, vascular disease, malignancy, infection, liver disease and hypovolaemia were all predictive of readmission; AKI was not; perhaps due to the high mortality rate in this group.. As a result, in patients who survive, those with AKI are less likely to be re-admitted probably as a combination of survival advantage, and potentially for some, because of palliative care packages which reduce readmission. In our study AKI did not increase 30 or 90 day readmission rates, with death included or excluded. Overall rate of readmission was not increased for the whole period of follow up.

Interventions which may impact death in the early follow up period and readmission have not been extensively researched but it may be relevant for nephrologists to be involved in early post-discharge care in patients who have had AKI. This may influence drug management, early identification of deterioration with rapid access to dialysis and more meticulous fluid management particularly relevant as early pulmonary oedema has been linked to readmission [43].

There is little literature discussing AKI and readmission rates but AKI has previously been reported as contributing to readmission rates with increased risk of readmission at 30, 60 and 90 days. However, the rate is much attenuated after adjustment and is only present at 30-days in the matched validation cohort. Additionally in Silver et al. the reported mortality rate at the index admission is only 2%, very low compared to data in our study and therefore may mean fundamentally different cohort. All patients were excluded in which a creatinine nadir was unable to be calculated.

Definition of AKI in the literature is variable since the considerable interest in this area has generated scoring systems which have been developed as this has been studied. We define AKI on the basis of the creatinine changes for KDIGO, superseding AKIN and RIFLE. Any definitions of AKI must include ability to easily identify patients using EPR which are likely become more ubiquitous with rapid adoption across the UK. Baseline creatinine is not necessarily available in acute admissions and we aimed to understand the impact of available hospital information at the point of patient treatment. Most patients have creatinine measured, some institutions use more complex measurements of renal functions, estimated GFR by various methods, CKI-EPI and others, but these are not yet standardised. Similarly urine output is not reliably collected in the majority of emergency admissions.

There are several limitations to this study. It is a single centre study, and although it is based in a large urban hospital, some of the hospital services are tertiary and therefore will over-represent relatively rarer disease states such as advanced liver disease. Although we have found AKI to be an independent risk factor it is often actually a marker of disease severity and may have as yet unidentified confounders within this study. Sicker patients are likely to have more creatinine measurements and therefore may skew the data. We have assumed that patients with one or less creatinine measurements did not have AKI. CKD rates are low, which is likely to be due to the exclusion of patients known to the renal service. CKD is a significant risk factor for AKI and patients who are sicker therefore be under nephrology and excluded from this analysis. This may result in fewer patients in this study with heavy comorbidity burdens, who may have been more likely to die or be readmitted after an AKI.

The study data collection ends in 2011, AKI has been increasingly discussed and management strategies therefore may have changed since the conclusion of this data collection. Readmission rates do not include patients admitted to other hospitals. KDIGO definition of AKI includes urine criteria and this is not utilised here as it is unreliably recorded in EPR. The impact of this is difficult to study as in practice urine output is poorly recorded in acute medical settings outside of high dependency beds.

## Conclusion

In a large, unselected hospital inpatient population, AKI identified during hospital admissions is associated with a high in-hospital mortality and this persists into the period up to 90 days post discharge and beyond. AKI is an independent risk factor in hospital and up to 90 days post discharge even when adjusted for co-morbidities, age, and CRP, and is associated with mortality after 90 days. There is a progressive relationship between CRP concentration, AKI and death which is poorly understood and there is a case for studying this further. Readmission rates were found to be unaffected by AKI.

It is important to note that many of the patients who are affected and who die with AKI are already under palliative care services and many have malignancy. This study may support the concept of interventions aimed at preventing AKI such as early nephrology review but post discharge interventions in terms of preventing mortality or 90 day readmission would need to be carefully targeted.

## Additional files

Additional file 1: Medications within respective drug classes. (DOCX 14 kb)

Additional file 2: ICD 10 diagnoses. (DOCX 15 kb)

Additional file 3: Integrated codes for composite of infections. (DOCX 14 kb)

Additional file 4: Cox regression for in-hospital mortality AKI stage and adjusted for age, gender, co-morbidity and CRP. (DOCX 19 kb)

Additional file 5: Cox regression for Post Discharge death after 90 Days for AKI and adjusted for age, gender, co-morbidity and CRP. (DOCX 20 kb)

Additional file 6: Cox regression for Post Discharge death after 90 Days for AKI stage and adjusted for age, gender, co-morbidity and CRP. (DOCX 14 kb)

Additional file 7: Odds Ratio for all-cause in hospital mortality associated with post index admission AKI. (DOCX 14 kb)

Additional file 8: Logistic regression for readmission within 90 days for AKI stage and adjusted for age, gender, co-morbidity and CRP. (DOCX 18 kb)

Additional file 9: Cox regression for time to readmission from 1st admission dependent on AKI, excluding patients who died, and adjusted for age, gender, co-morbidity and CRP. (DOCX 21 kb)

Additional file 10: Comparison of comorbidities between the differing populations of patients: those who died in the first admission, those who survived and were readmitted and those who survived and weren’t readmitted. (DOCX 15 kb)

Additional file 11: Univariable and restricted multivariable analyses by Cox regression, for CRP effect on inpatient mortality, 90 day mortality and mortality beyond 90 days. (DOCX 33 kb)

## Abbreviations

AKI: Acute kidney injury
CI: Confidence intervals
CRP: C-reactive protein
EPR: Electronic patient record
HES: Hospital Episode Statistics
HR: Hazard ratios
ICD10: International Statistical Classification of Diseases and Related Health Problems 10
ICU: Intensive care unit
IQR: Interquartile range
KDIGO: Kidney disease improving global outcomes
LoS: Length of stay
OR: Odds ratio
RRT: Renal replacement therapy
SD: Standard deviation
