# Supplementary material for: Hospital acquired Acute Kidney Injury is associated with increased mortality but not increased readmission rates in a UK acute hospital
Source: BMC Nephrol. 2017 Oct 20;18:317. doi: 10.1186/s12882-017-0729-9 (PMC5651577; doi:10.1186/s12882-017-0729-9)
Supplement: Supplementary file 1 — Medications within respective drug classes. (DOCX 14 kb) [file 12882_2017_729_MOESM1_ESM.docx]

**Additional File 1: medications within respective drug classes**

| The following medications were prescribed within the respective drug classes  *Angiotensin-converting enzyme inhibitors and angiotensin II receptor antagonists*: Captopril, Imidapril, Lisinopril, Perindopril, Ramipril, Trandolapril, Candesartan, Eprosartan, Irbesartan, Losartan, Telmisartan & Valsartan.  *Non-steroidal anti-inflammatory drugs:* Aceclofenac, Acemetacin, Celecoxib, Dexibuprofen, Dexketoprofen, Diclofenac, Etodolac, Etoricoxib, *Etoricoximab*, Fenbufen (now deleted from formulary), Flurbiprofen, Ibuprofen, Indometacin, Ketoprofen, Lederfen (not recognised), Mefenamic Acid, Meloxicam, Naproxen, Piroxicam, Sulindac, Tenoxicam & Tiaprofenic Acid.  *Antimicrobials:* Aciclovir, Albendazole, AmBisome (from amphotericin), Amikacin, Amoxicillin, Azithromycin, Aztreonam, Benzylpenicillin, Cefadroxil, Cefalexin, Cefixime, Cefotaxime, Ceftazidime, Ceftriaxone, Cefuroxime, Ciprofloxacin, Clarithromycin, Clindamycin, Co-amoxiclav, Colistimethate, Co-trimoxazole, Dapsone, Daptomycin, Doxycycline, Ertapenem, Erythromycin, Flucloxacillin, Fluconazole, Fungizone (from amphotericin), Ganciclovir, Gentamicin, Imipenem with Cilastatin, Isoniazid, Itraconazole, Levofloxacin, Linezolid, Lymecycline, Meropenem, Micafungin (TENPIN) Trial Drug, Minocycline, Moxifloxacin, Neomycin, Nitrofurantoin, Norfloxacin, Ofloxacin, Oxytetracycline, Penicillin V, Piperacillin with Tazobactam, Pyrazinamide, Pyrimethamine, Rifabutin, Rifampicin, Sodium Fusidate, Streptomycin, Sulfadiazine, Teicoplanin, Tetracycline, Tigecycline, Tobramycin, Trimethoprim & Vancomycin.  *Diuretics:* Amiloride, Bendroflumethiazide, Bumetanide, Chlortalidone, Cyclopenthiazide, Eplerenone, Furosemide, Indapamide, Metolazone, Spironolactone, Torasemide, Triamterene & Xipamide |
| --- |
