# Supplementary material for: Hospital acquired Acute Kidney Injury is associated with increased mortality but not increased readmission rates in a UK acute hospital
Source: BMC Nephrol. 2017 Oct 20;18:317. doi: 10.1186/s12882-017-0729-9 (PMC5651577; doi:10.1186/s12882-017-0729-9)
Supplement: Supplementary file 2 — ICD 10 diagnoses. (DOCX 15 kb) [file 12882_2017_729_MOESM2_ESM.docx]

**Additional File 2: ICD 10 diagnoses**

| ICD 10 diagnoses coded at discharge were combined to identify specific groups at high risk of AKI.   1. Diabetes mellitus (E10-E14) 2. Hypertension (I10 – I15) 3. Heart failure (I50) 4. Peripheral vascular disease (I70-I79) 5. Ischemic heart disease (I20-25) 6. Malignancy excluding non-melanoma skin cancer (C00-C43, C45-C97) 7. Gastrointestinal bleed and hypovolemia: hematemesis (K92.0), melena (K92.1), gastrointestinal haemorrhage (K92.2), volume depletion (E86, R57.1, T81.1) 8. Infection and pancreatitis: Meningitis (A17, A20.3, A32.1, A39, G96.1, G00, G01, G042), streptococcal sepsis (A40), pancreatitis (K85), cholangitis (K80.3, K83.0), peritonitis (K35, K65), endocarditis (I38, I33, I38, I39), fasciitis (M72.5) other sepsis (A41), other bacterial infection (A04), urinary tract infection (N39.0), acute cystitis (N30). 9. Liver disease (K70-77). |
| --- |
