# Supplementary material for: Hospital acquired Acute Kidney Injury is associated with increased mortality but not increased readmission rates in a UK acute hospital
Source: BMC Nephrol. 2017 Oct 20;18:317. doi: 10.1186/s12882-017-0729-9 (PMC5651577; doi:10.1186/s12882-017-0729-9)
Supplement: Supplementary file 3 — Integrated codes for composite of infections. (DOCX 14 kb) [file 12882_2017_729_MOESM3_ESM.docx]

**Additional File 3: Integrated codes for sepsis**

| This progressive relationship between CRP concentration and mortality emerged despite inclusion in the model of post-admission AKI and a variable that integrated many diagnoses associated with the acute inflammatory response including various forms of ‘sepsis’.  Diagnoses (ICD10 code) integrated into this single parameter were: Meningitis (A17, A20.3, A32.1, A39, G96.1, G00, G01, G042), streptococcal sepsis (A40), pancreatitis (K85), cholangitis (K80.3, K83.0), peritonitis (K35, K65), endocarditis (I38, I33, I38, I39), fasciitis (M72.5) other sepsis (A41), other bacterial infection (A04), urinary tract infection (N39.0), acute cystitis (N30) |
| --- |
