# Supplementary material for: Hospital acquired Acute Kidney Injury is associated with increased mortality but not increased readmission rates in a UK acute hospital
Source: BMC Nephrol. 2017 Oct 20;18:317. doi: 10.1186/s12882-017-0729-9 (PMC5651577; doi:10.1186/s12882-017-0729-9)
Supplement: Supplementary file 4 — Cox regression for in-hospital mortality AKI stage and adjusted for age, gender, co-morbidity and CRP. (DOCX 19 kb) [file 12882_2017_729_MOESM4_ESM.docx]

Replacement for

Additional file 4: Cox regression for in-hospital mortality AKI stage and adjusted for age, gender, co-morbidity and CRP

|  |  | Hazard Ratio | 95% Confidence intervals | | P value |
| --- | --- | --- | --- | --- | --- |
|  |  |  | Lower | Upper |  |
|  | AKI stage 1 | 1.469 | 1.295 | 1.667 | <0.001 |
|  | AKI stage 2 | 1.970 | 1.689 | 2.297 | <0.001 |
|  | AKI stage 3 | 1.270 | 0.987 | 1.634 | 0.063 |
|  | Male gender | 1.024 | 0.950 | 1.103 | 0.531 |
|  | Age (reference 18-35 years) | | | | |
|  | Age 36-45 | 1.943 | 1.251 | 3.016 | 0.003 |
|  | Age 46-55 | 4.118 | 2.833 | 5.986 | <0.001 |
|  | Age 56-65 | 5.511 | 3.855 | 7.877 | <0.001 |
|  | Age 66-75 | 8.655 | 6.113 | 12.254 | <0.001 |
|  | Age >75 | 16.887 | 12.041 | 23.682 | <0.001 |
|  | Diabetes Mellitus | 0.964 | 0.872 | 1.067 | 0.482 |
|  | Hypertension | 0.740 | 0.682 | 0.802 | <0.001 |
|  | Ischaemic Heart Disease | 0.775 | 0.669 | 0.899 | 0.001 |
|  | Heart Failure | 1.692 | 1.527 | 1.876 | <0.001 |
|  | Vascular Disease | 1.476 | 1.213 | 1.796 | <0.001 |
|  | Malignancy | 1.879 | 1.724 | 2.048 | <0.001 |
|  | Composite of Infection | 0.813 | 0.740 | 0.894 | <0.001 |
|  | Liver Disease | 2.644 | 2.288 | 3.054 | <0.001 |
|  | Composite of GI Blood Loss or Hypovolaemia | 1.640 | 1.463 | 1.838 | <0.001 |
|  | CRP (referenced to CRP<11) |  | |  | |
|  | Unmeasured | 1.220 | 0.907 | 1.642 | 0.189 |
|  | 11-20 | 2.085 | 1.568 | 2.772 | <0.001 |
|  | 21-30 | 2.228 | 1.667 | 2.976 | <0.001 |
|  | 31-40 | 2.640 | 1.986 | 3.509 | <0.001 |
|  | 41-50 | 2.381 | 1.767 | 3.210 | <0.001 |
|  | 51-60 | 2.234 | 1.639 | 3.045 | <0.001 |
|  | 61-70 | 3.027 | 2.256 | 4.061 | <0.001 |
|  | 71-80 | 2.647 | 1.967 | 3.562 | <0.001 |
|  | 81-90 | 3.215 | 2.384 | 4.335 | <0.001 |
|  | 91-100 | 3.064 | 2.433 | 3.861 | <0.001 |
|  | 101-150 | 3.652 | 2.885 | 4.625 | <0.001 |
|  | 151-200 | 4.034 | 3.191 | 5.101 | <0.001 |
|  | 201-250 | 4.073 | 3.173 | 5.228 | <0.001 |
|  | 251-300 | 4.421 | 3.425 | 5.707 | <0.001 |
|  | 301-350 | 4.162 | 3.023 | 5.729 | <0.001 |
|  | 351-400 | 2.961 | 2.369 | 3.701 | <0.001 |
|  | >400 | 5.925 | 4.470 | 7.854 | <0.001 |
