# Supplementary material for: Hospital acquired Acute Kidney Injury is associated with increased mortality but not increased readmission rates in a UK acute hospital
Source: BMC Nephrol. 2017 Oct 20;18:317. doi: 10.1186/s12882-017-0729-9 (PMC5651577; doi:10.1186/s12882-017-0729-9)
Supplement: Supplementary file 5 — Cox regression for Post Discharge death after 90 Days for AKI and adjusted for age, gender, co-morbidity and CRP. (DOCX 20 kb) [file 12882_2017_729_MOESM5_ESM.docx]

**Additional File 5**

**Cox regression for Post Discharge death after 90 Days for AKI stage and adjusted for age, gender, co-morbidity (Diabetes Mellitus; hypertension; heart failure; vascular disease; malignancy; composite of infection; composite of GI blood loss or hypovolemia)**  **and CRP**

|  |  |  |  |  | Odds Ratio | Confidence Intervals | | p |
| --- | --- | --- | --- | --- | --- | --- | --- | --- |
|  |  |  |  |  |  | Lower | Upper |  |
| AKI |  |  |  |  | 2.915 | 2.701 | 3.146 | <0.001 |
| Male gender |  |  |  |  | 1.078 | 1.032 | 1.126 | 0.001 |
| Age 46-55 |  |  |  |  | 2.335 | 1.911 | 2.853 | <0.001 |
| Age 56-65 |  |  |  |  | 4.783 | 4.011 | 5.704 | <0.001 |
| Age 66-75 |  |  |  |  | 8.104 | 6.861 | 9.573 | <0.001 |
| Age >75 |  |  |  |  | 13.638 | 11.595 | 16.042 | <0.001 |
| Diabetes mellitus |  |  |  |  | 1.089 | 1.026 | 1.156 | 0.005 |
| Hypertension |  |  |  |  | 0.762 | 0.726 | 0.800 | <0.001 |
| Ischemic Heart Disease |  |  |  |  | 0.936 | 0.864 | 1.013 | 0.100 |
| Vascular disease |  |  |  |  | 1.323 | 1.134 | 1.543 | <0.001 |
| Cardiac Failure |  |  |  |  | 1.547 | 1.425 | 1.679 | <0.001 |
| Malignancy |  |  |  |  | 5.015 | 4.753 | 5.292 | <0.001 |
| Composite of Infection |  |  |  |  | 1.161 | 1.080 | 1.249 | <0.001 |
| Liver Disease |  |  |  |  | 1.914 | 1.689 | 2.169 | <0.001 |
| Composite of GI blood loss or hypovolaemia |  |  |  |  | 1.221 | 1.097 | 1.359 | <0.001 |
| CRP |  |  |  |  |  |  |  |  |
| unmeasured |  |  |  |  | 0.749 | 0.699 | 0.802 | <0.001 |
| 11-20 |  |  |  |  | 1.161 | 1.058 | 1.273 | 0.002 |
| 21-30 |  |  |  |  | 1.255 | 1.127 | 1.397 | <0.001 |
| 31-40 |  |  |  |  | 1.251 | 1.111 | 1.408 | <0.001 |
| 41-50 |  |  |  |  | 1.231 | 1.081 | 1.401 | 0.002 |
| 51-60 |  |  |  |  | 1.055 | 0.918 | 1.213 | 0.450 |
| 61-70 |  |  |  |  | 1.170 | 1.012 | 1.352 | 0.034 |
| 71-80 |  |  |  |  | 1.286 | 1.105 | 1.496 | 0.001 |
| 81-90 |  |  |  |  | 1.123 | 0.963 | 1.309 | 0.140 |
| 91-100 |  |  |  |  | 1.318 | 1.123 | 1.546 | 0.001 |
| 101-150 |  |  |  |  | 1.139 | 1.031 | 1.259 | 0.011 |
| 151-200 |  |  |  |  | 1.136 | 1.012 | 1.275 | 0.031 |
| 201-250 |  |  |  |  | 1.015 | 0.898 | 1.147 | 0.820 |
| 251-300 |  |  |  |  | 0.926 | 0.789 | 1.086 | 0.340 |
| 301-350 |  |  |  |  | 0.713 | 0.580 | 0.876 | 0.001 |
| 350-400 |  |  |  |  | 0.942 | 0.714 | 1.241 | 0.670 |
| >400 |  |  |  |  | 0.870 | 0.663 | 1.142 | 0.320 |
