# Supplementary material for: Hospital acquired Acute Kidney Injury is associated with increased mortality but not increased readmission rates in a UK acute hospital
Source: BMC Nephrol. 2017 Oct 20;18:317. doi: 10.1186/s12882-017-0729-9 (PMC5651577; doi:10.1186/s12882-017-0729-9)
Supplement: Supplementary file 6 — Cox regression for Post Discharge death after 90 Days for AKI stage and adjusted for age, gender, co-morbidity and CRP. (DOCX 14 kb) [file 12882_2017_729_MOESM6_ESM.docx]

**Additional File 6**

**Odds Ratio for all-cause in hospital mortality associated with post index admission AKI**

|  | Unadjusted | Adjusted | | |
| --- | --- | --- | --- | --- |
|  |  | Age & Sex | Age, Sex & Co-morbidity | Age, Sex, Co-morbidity & CRP |
| No AKI | ref | ref | ref | ref |
| AKI | 14.8 (13.2-16.5) | 10.2 (9.1-11.5) | 7.0 (6.8-7.9) | 4.0 (3.5-4.6) |
| AKI 1 | 12.2 (10.6-14.1) | 7.8 (6.7-9.1) | 5.5 (4.6-6.4) | 3.4 (2.9-4.0) |
| AKI 2 | 22.2 (18.3-26.9) | 17.2 (13.9-22.2) | 11.9 (9.5-14.9) | 6.1 (4.9-7.7) |
| AKI 3 | 14.4 (10.6-19.4) | 12.1 (8.7-16.7) | 6.9 (4.9-9.7) | 3.4 (2.4-4.8) |
