# Supplementary material for: Hospital acquired Acute Kidney Injury is associated with increased mortality but not increased readmission rates in a UK acute hospital
Source: BMC Nephrol. 2017 Oct 20;18:317. doi: 10.1186/s12882-017-0729-9 (PMC5651577; doi:10.1186/s12882-017-0729-9)
Supplement: Supplementary file 7 — Odds Ratio for all-cause in hospital mortality associated with post index admission AKI. (DOCX 14 kb) [file 12882_2017_729_MOESM7_ESM.docx]

**Additional File 7**

**Odds Ratio for all-cause 90 day mortality after discharge following index admission AKI**

|  | Unadjusted | Adjusted | | |
| --- | --- | --- | --- | --- |
|  |  | Age & Sex | Age, Sex & Co-morbidity | Age, Sex, Co-morbidity & CRP |
| No AKI | ref | ref | ref | ref |
| AKI | 4.1 (3.4-4.8) | 2.6 (2.2-3.1) | 2.1 (1.7-2.5) | 1.5 (1.3-1.9) |
| AKI 1 | 3.7 (2.9-4.6) | 2.3 (1.8-2.8) | 1.7 (1.4-2.2) | 1.3 (1.0-1.7) |
| AKI 2 | 4.2 (3.0-6.0) | 2.9 (2.0-4.1) | 2.3 (1.6-3.4) | 1.7 (1.1-2.5) |
| AKI 3 | 6.0 (3.9-9.2) | 4.8 (3.1-7.6) | 3.7 (2.3-6.0) | 2.6 (1.6-4.2) |
