# Supplementary material for: Hospital acquired Acute Kidney Injury is associated with increased mortality but not increased readmission rates in a UK acute hospital
Source: BMC Nephrol. 2017 Oct 20;18:317. doi: 10.1186/s12882-017-0729-9 (PMC5651577; doi:10.1186/s12882-017-0729-9)
Supplement: Supplementary file 8 — Logistic regression for readmission within 90 days for AKI stage and adjusted for age, gender, co-morbidity and CRP. (DOCX 18 kb) [file 12882_2017_729_MOESM8_ESM.docx]

Replacement for

Additional file 8: Cox regression for readmission within 90 days for AKI stage and adjusted for age, gender, co-morbidity and CRP

|  |  | Hazard Ratio | 95% Confidence intervals | | P value |
| --- | --- | --- | --- | --- | --- |
|  |  |  | Lower | Upper |  |
|  | AKI stage 1 | 0.984 | 0.816 | 1.185 | 0.862 |
|  | AKI stage 2 | 0.916 | 0.666 | 1.259 | 0.589 |
|  | AKI stage 3 | 0.586 | 0.339 | 1.013 | 0.056 |
|  | Male gender | 0.932 | 0.891 | 0.975 | 0.002 |
|  | Age (reference 18-35 years) | | | | |
|  | Age 36-45 | 1.076 | 0.983 | 1.178 | 0.113 |
|  | Age 46-55 | 1.254 | 1.152 | 1.366 | <0.001 |
|  | Age 56-65 | 1.261 | 1.158 | 1.373 | <0.001 |
|  | Age 66-75 | 1.441 | 1.325 | 1.567 | <0.001 |
|  | Age >75 | 1.782 | 1.655 | 1.920 | <0.001 |
|  | Diabetes Mellitus | 1.186 | 1.109 | 1.269 | <0.001 |
|  | Hypertension | 0.939 | 0.888 | 0.994 | 0.029 |
|  | Ischaemic Heart Disease | 1.107 | 1.011 | 1.212 | 0.028 |
|  | Heart Failure | 1.372 | 1.233 | 1.526 | <0.001 |
|  | Vascular Disease | 1.195 | 0.984 | 1.451 | 0.072 |
|  | Malignancy | 2.111 | 1.975 | 2.257 | <0.001 |
|  | Composite of Infection | 1.149 | 1.057 | 1.249 | 0.001 |
|  | Liver Disease | 1.785 | 1.564 | 2.037 | <0.001 |
|  | Composite of GI Blood Loss or Hypovolaemia | 1.260 | 1.117 | 1.421 | <0.001 |
|  | CRP (referenced to CRP<11) |  | |  | |
|  | Unmeasured | 1.009 | 0.914 | 1.114 | 0.857 |
|  | 11-20 | 1.068 | 0.949 | 1.203 | 0.274 |
|  | 21-30 | 1.050 | 0.920 | 1.199 | 0.467 |
|  | 31-40 | 1.107 | 0.960 | 1.276 | 0.162 |
|  | 41-50 | 1.138 | 0.982 | 1.319 | 0.085 |
|  | 51-60 | 1.093 | 0.931 | 1.283 | 0.277 |
|  | 61-70 | 1.138 | 0.963 | 1.345 | 0.129 |
|  | 71-80 | 1.068 | 0.898 | 1.270 | 0.458 |
|  | 81-90 | 0.932 | 0.764 | 1.136 | 0.485 |
|  | 91-100 | 1.068 | 0.958 | 1.192 | 0.235 |
|  | 101-150 | 1.087 | 0.957 | 1.235 | 0.200 |
|  | 151-200 | 1.097 | 0.957 | 1.256 | 0.183 |
|  | 201-250 | 1.090 | 0.920 | 1.293 | 0.319 |
|  | 251-300 | 0.768 | 0.614 | 0.959 | 0.020 |
|  | 301-350 | 1.335 | 1.026 | 1.736 | 0.032 |
|  | 351-400 | 0.843 | 0.788 | 0.902 | <0.001 |
|  | >400 | 1.047 | .792 | 1.386 | 0.746 |
