# Supplementary material for: Hospital acquired Acute Kidney Injury is associated with increased mortality but not increased readmission rates in a UK acute hospital
Source: BMC Nephrol. 2017 Oct 20;18:317. doi: 10.1186/s12882-017-0729-9 (PMC5651577; doi:10.1186/s12882-017-0729-9)
Supplement: Supplementary file 9 — Cox regression for time to readmission from 1st admission dependent on AKI, excluding patients who died, and adjusted for age, gender, co-morbidity and CRP. (DOCX 21 kb) [file 12882_2017_729_MOESM9_ESM.docx]

**Additional File 9**

**Cox regression for time to readmission from 1^st^ admission dependent on AKI, excluding patients who died, and adjusted for age, gender, co-morbidity (Diabetes Mellitus; hypertension; heart failure; vascular disease; malignancy; composite of infection; composite of GI blood loss or hypovolemia)**  **and CRP**

|  |  |  |  |  | Hazard Ratio | 95.0% CI for Exp(B) | | p value |
| --- | --- | --- | --- | --- | --- | --- | --- | --- |
|  |  |  |  |  |  | Lower | Upper |  |
| AKI stage1 |  |  |  |  | .830 | .728 | .946 | .005 |
| AKI stage2 |  |  |  |  | .757 | .606 | .947 | .015 |
| AKI stage 3 |  |  |  |  | .588 | .410 | .843 | .004 |
| Male gender |  |  |  |  | .889 | .864 | .915 | <0.001 |
| Age |  |  |  |  | 1.203 | 1.152 | 1.256 | <0.001 |
| Age 36-45 |  |  |  |  | .922 | .890 | .955 | <0.001 |
| Age 46-55 |  |  |  |  | 1.238 | 1.172 | 1.306 | <0.001 |
| Age 56-65 |  |  |  |  | 1.317 | 1.229 | 1.411 | <0.001 |
| Age 66-75 |  |  |  |  | 1.363 | 1.213 | 1.532 | <0.001 |
| Age >75 |  |  |  |  | 1.877 | 1.786 | 1.972 | <0.001 |
| Diabetes Mellitus |  |  |  |  | 1.186 | 1.123 | 1.251 | <0.001 |
| Hypertension |  |  |  |  | 1.779 | 1.623 | 1.951 | <0.001 |
| Ischaemic Heart Disease |  |  |  |  | 1.227 | 1.133 | 1.329 | <0.001 |
| Heart Failure |  |  |  |  |  |  |  | <0.001 |
| Vascular Disease |  |  |  |  | 1.142 | 1.075 | 1.214 | <0.001 |
| Malignancy |  |  |  |  | 1.434 | 1.355 | 1.518 | <0.001 |
| Composite of Infection |  |  |  |  | 1.523 | 1.440 | 1.612 | <0.001 |
| Liver Disease |  |  |  |  | 1.853 | 1.754 | 1.958 | <0.001 |
| Composite of GI Blood Loss or Hypovolaemia |  |  |  |  | 2.765 | 2.634 | 2.903 | <0.001 |
| CRP |  |  |  |  |  |  |  | <0.001 |
| Unmeasured |  |  |  |  | .848 | .813 | .885 | <0.001 |
| 11-20 |  |  |  |  | 1.029 | .966 | 1.096 | .377 |
| 21-30 |  |  |  |  | 1.064 | .985 | 1.148 | .113 |
| 31-40 |  |  |  |  | 1.068 | .980 | 1.163 | .132 |
| 41-50 |  |  |  |  | 1.075 | .978 | 1.181 | .132 |
| 51-60 |  |  |  |  | 1.036 | .938 | 1.144 | .488 |
| 61-70 |  |  |  |  | 1.011 | .908 | 1.126 | .837 |
| 71-80 |  |  |  |  | 1.009 | .900 | 1.131 | .877 |
| 81-90 |  |  |  |  | .954 | .848 | 1.074 | .436 |
| 91-100 |  |  |  |  | .975 | .861 | 1.105 | .694 |
| 101-150 |  |  |  |  | 1.005 | .935 | 1.081 | .886 |
| 151-200 |  |  |  |  | .982 | .901 | 1.070 | .679 |
| 201-250 |  |  |  |  | 1.005 | .919 | 1.100 | .909 |
| 251-300 |  |  |  |  | .990 | .884 | 1.109 | .865 |
| 301-350 |  |  |  |  | .775 | .671 | .895 | .001 |
| 350-400 |  |  |  |  | 1.093 | .907 | 1.319 | .350 |
| >400 |  |  |  |  | .929 | .768 | 1.124 | .448 |
