# Supplementary material for: Hospital acquired Acute Kidney Injury is associated with increased mortality but not increased readmission rates in a UK acute hospital
Source: BMC Nephrol. 2017 Oct 20;18:317. doi: 10.1186/s12882-017-0729-9 (PMC5651577; doi:10.1186/s12882-017-0729-9)
Supplement: Supplementary file 10 — Comparison of comorbidities between the differing populations of patients: those who died in the first admission, those who survived and were readmitted and those who survived and weren’t readmitted. (DOCX 15 kb) [file 12882_2017_729_MOESM10_ESM.docx]

Additional File 10: Comparison of comorbidities between the differing populations of patients: those who died in the first admission, those who survived and were readmitted and those who survived and weren’t readmitted.

|  | Died in index admission cf Survived and readmitted (Fishers Exact) | | | Died in index admission cf Survived and not readmitted  (Fishers Exact) | | | Survived and readmitted cf Survived and not readmitted  (Fishers Exact) | | | Comparison of all three populations (Chi Squared) |
| --- | --- | --- | --- | --- | --- | --- | --- | --- | --- | --- |
|  | Died in index admission  N=2918 | Survived and readmitted  N=4297 | p | Died in index admission  N=2918 | Survived and not readmitted  N=65801 | p | Survived and readmitted  N=4297 | Survived and not readmitted  N=65801 | p | p |
| Diabetes mellitus n (%) | 472 (16) | 490 (11) | <0.001 | 472 (16) | 6609 (10) | <0.001 | 490 (13) | 6609 (10) | 0.005 | <0.001 |
| Hypertension n (%) | 896 (31) | 927 (22) | <0.001 | 896 (31) | 13680 (21) | <0.001 | 927 (22) | 13680 (21) | 0.222 | <0.001 |
| Ischaemic heart disease n (%) | 196 (7) | 260 (6) | 0.257 | 196 (7) | 3546 (5) | 0.02 | 260 (6) | 3545 (5) | 0.064 | 0.002 |
| Heart failure n (%) | 467 (16) | 162 (4) | <0.001 | 467 (16) | 1831 (3) | <0.001 | 162 (4) | 1831 (3) | <0.001 | <0.001 |
| Vascular disease n (%) | 107 (4) | 431 (1) | <0.001 | 107 (37) | 612 (1) | <0.001 | 431 (1) | 612 (1) | 0.631 | <0.001 |
| Malignancy n (%) | 740 (25) | 555 (13) | <0.001 | 740 (25) | 4223 (6) | <0.001 | 555 (13) | 4223 (6) | <0.001 | <0.001 |
| Composite of Infection n (%) | 614 (21) | 311 (7) | <0.001 | 614 (21) | 3995 (6) | <0.001 | 311 (7) | 3995 (6) | 0.02 | <0.001 |
| Liver Disease | 215 (7) | 111 (3) | <0.001 | 215 (7) | 1025 (2) | <0.001 | 111 (3) | 1025 (2) | <0.001 | <0.001 |
| GI blood loss | 349 (12) | 159 (4) | <0.001 | 349 (12) | 1550 (2) | <0.001 | 159 (4) | 1550 (2) | <0.001 | <0.001 |
