# Supplementary material for: Hospital acquired Acute Kidney Injury is associated with increased mortality but not increased readmission rates in a UK acute hospital
Source: BMC Nephrol. 2017 Oct 20;18:317. doi: 10.1186/s12882-017-0729-9 (PMC5651577; doi:10.1186/s12882-017-0729-9)
Supplement: Supplementary file 11 — Univariable and restricted multivariable analyses by Cox regression, for CRP effect on inpatient mortality, 90 day mortality and mortality beyond 90 days. (DOCX 33 kb) [file 12882_2017_729_MOESM11_ESM.docx]

Additional file 11: univariable and restricted multivariable analyses by Cox regression, for CRP effect on inpatient mortality, 90 day mortality and mortality beyond 90 days

Table 11a: Univariable analysis for CRP (and multivariable including only AKI) for inpatient mortality

CRP and inpatient mortality is closely correlated:

|  | Hazard ratio | Confidence Intervals | | p value |
| --- | --- | --- | --- | --- |
| CRP (referenced to CRP<11) |  | l |  |  |
| Unmeasured | 1.407 | 1.046 | 1.893 | .024 |
| 11-20 | 2.546 | 1.915 | 3.385 | .000 |
| 21-30 | 2.836 | 2.124 | 3.787 | .000 |
| 31-40 | 3.622 | 2.726 | 4.812 | .000 |
| 41-50 | 3.059 | 2.270 | 4.120 | .000 |
| 51-60 | 2.957 | 2.171 | 4.026 | .000 |
| 61-70 | 3.938 | 2.937 | 5.282 | .000 |
| 71-80 | 3.748 | 2.787 | 5.040 | .000 |
| 81-90 | 4.289 | 3.183 | 5.778 | .000 |
| 91-100 | 4.483 | 3.564 | 5.639 | .000 |
| 101-150 | 5.358 | 4.240 | 6.772 | .000 |
| 151-200 | 5.864 | 4.646 | 7.403 | .000 |
| 201-250 | 5.601 | 4.373 | 7.175 | .000 |
| 251-300 | 6.092 | 4.734 | 7.840 | .000 |
| 301-350 | 5.280 | 3.844 | 7.251 | .000 |
| 351-400 | 2.627 | 2.102 | 3.283 | .000 |
| >400 | 7.564 | 5.734 | 9.977 | .000 |

Table 11b: When adjusted for AKI the correlation is still strong:

|  | Hazard ratio | Confidence Intervals | | p value |
| --- | --- | --- | --- | --- |
| AKI | 1.826 | 1.654 | 2.015 | .000 |
| CRP (referenced to CRP<11) |  | | | |
| Unmeasured | 1.379 | 1.025 | 1.856 | .034 |
| 11-20 | 2.506 | 1.885 | 3.331 | .000 |
| 21-30 | 2.737 | 2.049 | 3.655 | .000 |
| 31-40 | 3.506 | 2.639 | 4.658 | .000 |
| 41-50 | 2.921 | 2.168 | 3.935 | .000 |
| 51-60 | 2.776 | 2.038 | 3.781 | .000 |
| 61-70 | 3.805 | 2.837 | 5.104 | .000 |
| 71-80 | 3.546 | 2.636 | 4.770 | .000 |
| 81-90 | 4.004 | 2.971 | 5.398 | .000 |
| 91-100 | 4.174 | 3.316 | 5.253 | .000 |
| 101-150 | 4.961 | 3.923 | 6.274 | .000 |
| 151-200 | 5.367 | 4.248 | 6.780 | .000 |
| 201-250 | 4.986 | 3.888 | 6.395 | .000 |
| 251-300 | 5.292 | 4.105 | 6.822 | .000 |
| 301-350 | 4.502 | 3.273 | 6.193 | .000 |
| 351-400 | 2.621 | 2.097 | 3.275 | .000 |
| >400 | 6.168 | 4.662 | 8.160 | .000 |

Table 11c: CRP effect on mortality is present at 90 days

|  | Hazard ratio | Confidence Intervals | | p value |
| --- | --- | --- | --- | --- |
| CRP (referenced to CRP<11) |  |  |  |  |
| Unmeasured | 1.344 | 1.268 | 1.424 | <0.001 |
| 11-20 | 1.601 | 1.499 | 1.710 | <0.001 |
| 21-30 | 1.775 | 1.652 | 1.906 | <0.001 |
| 31-40 | 1.870 | 1.734 | 2.017 | <0.001 |
| 41-50 | 1.816 | 1.675 | 1.970 | <0.001 |
| 51-60 | 1.984 | 1.825 | 2.156 | <0.001 |
| 61-70 | 2.091 | 1.919 | 2.279 | <0.001 |
| 71-80 | 2.034 | 1.857 | 2.228 | <0.001 |
| 81-90 | 2.050 | 1.862 | 2.255 | <0.001 |
| 91-100 | 2.119 | 1.999 | 2.246 | <0.001 |
| 101-150 | 2.170 | 2.029 | 2.321 | <0.001 |
| 151-200 | 2.181 | 2.029 | 2.345 | <0.001 |
| 201-250 | 2.069 | 1.893 | 2.261 | <0.001 |
| 251-300 | 1.831 | 1.648 | 2.035 | <0.001 |
| 301-350 | 1.632 | 1.379 | 1.933 | <0.001 |
| 351-400 | .668 | .639 | .698 | <0.001 |
| >400 | 1.768 | 1.511 | 2.069 | <0.001 |

Table 11d: CRP effect on mortality is present at 90 days when adjusted for AKI

|  | Hazard ratio | Confidence Intervals | | p value |
| --- | --- | --- | --- | --- |
| AKI | 1.376 | 1.277 | 1.484 | <0.001 |
| CRP (referenced to CRP<11) |  |  |  |  |
| Unmeasured | 1.341 | 1.266 | 1.421 | <0.001 |
| 11-20 | 1.594 | 1.492 | 1.703 | <0.001 |
| 21-30 | 1.763 | 1.641 | 1.894 | <0.001 |
| 31-40 | 1.854 | 1.720 | 2.000 | <0.001 |
| 41-50 | 1.806 | 1.665 | 1.958 | <0.001 |
| 51-60 | 1.966 | 1.808 | 2.137 | <0.001 |
| 61-70 | 2.073 | 1.902 | 2.259 | <0.001 |
| 71-80 | 2.010 | 1.835 | 2.201 | <0.001 |
| 81-90 | 2.027 | 1.842 | 2.230 | <0.001 |
| 91-100 | 2.090 | 1.972 | 2.216 | <0.001 |
| 101-150 | 2.135 | 1.996 | 2.283 | <0.001 |
| 151-200 | 2.135 | 1.986 | 2.295 | <0.001 |
| 201-250 | 2.013 | 1.841 | 2.201 | <0.001 |
| 251-300 | 1.776 | 1.598 | 1.974 | <0.001 |
| 301-350 | 1.569 | 1.325 | 1.858 | <0.001 |
| 351-400 | .669 | .640 | .699 | <0.001 |
| >400 | 1.688 | 1.441 | 1.976 | <0.001 |

Table 11e: CRP effect on mortality is present beyond 90 days

|  | Hazard ratio | Confidence Intervals | | p value |
| --- | --- | --- | --- | --- |
| CRP (referenced to CRP<11) |  |  |  |  |
| Unmeasured | 1.370 | 1.293 | 1.451 | <0.001 |
| 11-20 | 1.644 | 1.539 | 1.756 | <0.001 |
| 21-30 | 1.821 | 1.695 | 1.957 | <0.001 |
| 31-40 | 1.952 | 1.810 | 2.105 | <0.001 |
| 41-50 | 1.878 | 1.732 | 2.037 | <0.001 |
| 51-60 | 2.058 | 1.893 | 2.237 | <0.001 |
| 61-70 | 2.203 | 2.021 | 2.401 | <0.001 |
| 71-80 | 2.121 | 1.937 | 2.323 | <0.001 |
| 81-90 | 2.157 | 1.960 | 2.374 | <0.001 |
| 91-100 | 2.223 | 2.097 | 2.356 | <0.001 |
| 101-150 | 2.259 | 2.113 | 2.416 | <0.001 |
| 151-200 | 2.207 | 2.054 | 2.373 | <0.001 |
| 201-250 | 2.149 | 1.966 | 2.349 | <0.001 |
| 251-300 | 1.969 | 1.772 | 2.188 | <0.001 |
| 301-350 | 1.672 | 1.412 | 1.980 | <0.001 |
| 351-400 | .626 | .599 | .655 | <0.001 |
| >400 | 1.838 | 1.571 | 2.151 | <0.001 |

Table 11f: CRP effect on mortality is present beyond 90 days when adjusted for AKI

|  | Hazard ratio | Confidence Intervals | | p value |
| --- | --- | --- | --- | --- |
|  |  |  |  |  |
| AKI | 1.500 | 1.392 | 1.617 | <0.001 |
| CRP (referenced to CRP<11) |  |  |  |  |
| Unmeasured | 1.366 | 1.289 | 1.447 | <0.001 |
| 11-20 | 1.636 | 1.532 | 1.748 | <0.001 |
| 21-30 | 1.808 | 1.683 | 1.942 | <0.001 |
| 31-40 | 1.931 | 1.790 | 2.082 | <0.001 |
| 41-50 | 1.866 | 1.721 | 2.024 | <0.001 |
| 51-60 | 2.035 | 1.872 | 2.213 | <0.001 |
| 61-70 | 2.180 | 2.000 | 2.376 | <0.001 |
| 71-80 | 2.090 | 1.909 | 2.290 | <0.001 |
| 81-90 | 2.129 | 1.934 | 2.343 | <0.001 |
| 91-100 | 2.187 | 2.062 | 2.318 | <0.001 |
| 101-150 | 2.213 | 2.070 | 2.367 | <0.001 |
| 151-200 | 2.150 | 2.000 | 2.311 | <0.001 |
| 201-250 | 2.075 | 1.898 | 2.268 | <0.001 |
| 251-300 | 1.891 | 1.701 | 2.103 | <0.001 |
| 301-350 | 1.593 | 1.345 | 1.887 | <0.001 |
| 351-400 | .628 | .600 | .656 | <0.001 |
| >400 | 1.735 | 1.482 | 2.032 | <0.001 |
